# Efficient energy transport in an organic semiconductor mediated by transient exciton delocalization

**DOI:** 10.1126/sciadv.abh4232

**Published:** 2021-08-04

**Authors:** Alexander J. Sneyd, Tomoya Fukui, David Paleček, Suryoday Prodhan, Isabella Wagner, Yifan Zhang, Jooyoung Sung, Sean M. Collins, Thomas J. A. Slater, Zahra Andaji-Garmaroudi, Liam R. MacFarlane, J. Diego Garcia-Hernandez, Linjun Wang, George R. Whittell, Justin M. Hodgkiss, Kai Chen, David Beljonne, Ian Manners, Richard H. Friend, Akshay Rao

**Affiliations:** 1 Department of Physics, Cavendish Laboratory, University of Cambridge, Cambridge CB3 0HE, UK.; 2 Department of Chemistry, University of Victoria, Victoria, BC V8P 5C2, Canada.; 3 School of Chemistry, University of Bristol, Bristol BS8 1TS, UK.; 4 Laboratory for Chemistry of Novel Materials, University of Mons, Mons 7000, Belgium.; 5 MacDiarmid Institute for Advanced Materials and Nanotechnology and School of Chemical and Physical Sciences, Victoria University of Wellington, Wellington 6010, New Zealand.; 6 School of Chemical and Process Engineering and School of Chemistry, University of Leeds, Leeds LS2 9JT, UK.; 7 Electron Physical Science Imaging Centre, Diamond Light Source Ltd., Oxfordshire OX11 0DE, UK.; 8 Center for Chemistry of Novel & High-Performance Materials, and Department of Chemistry, Zhejiang University, Hangzhou 310027, China.; 9 Robinson Research Institute, Faculty of Engineering, Victoria University of Wellington, Wellington 6012, New Zealand.; 10 The Dodd-Walls Centre for Photonic and Quantum Technologies, Dunedin 9016, New Zealand.

## Abstract

Efficient energy transport is desirable in organic semiconductor (OSC) devices. However, photogenerated excitons in OSC films mostly occupy highly localized states, limiting exciton diffusion coefficients to below ~10^−2^ cm^2^/s and diffusion lengths below ~50 nm. We use ultrafast optical microscopy and nonadiabatic molecular dynamics simulations to study well-ordered poly(3-hexylthiophene) nanofiber films prepared using living crystallization-driven self-assembly, and reveal a highly efficient energy transport regime: transient exciton delocalization, where energy exchange with vibrational modes allows excitons to temporarily re-access spatially extended states under equilibrium conditions. We show that this enables exciton diffusion constants up to 1.1 ± 0.1 cm^2^/s and diffusion lengths of 300 ± 50 nm. Our results reveal the dynamic interplay between localized and delocalized exciton configurations at equilibrium conditions, calling for a re-evaluation of exciton dynamics and suggesting design rules to engineer efficient energy transport in OSC device architectures not based on restrictive bulk heterojunctions.

## Teaser

Precisely tuning an organic semiconductor’s crystalline order allows exciton transport to proceed 2-3 orders of magnitude faster.

## INTRODUCTION

The efficient transport of energy in the form of excitons is crucial to the functioning of light-harvesting devices based on organic semiconductors (OSCs) as well as naturally occurring light-harvesting complexes (LHCs). In the former, it allows excitons generated in the material’s bulk to reach charge-generating heterojunctions, and in the latter, it allows energy to reach reaction centers. However, strong exciton-phonon couplings and electron-hole Coulomb interactions cause the localization of exciton wave functions in OSCs, and current models consider the physics of OSCs to be dominated by these localized states. Energy transport, in particular, is generally considered through the framework of Förster resonance energy transfer (FRET), where localized excitons hop incoherently from site to site (*1*–*3*). This mechanism naturally limits the distance that excitons can travel (*3*). In LHCs, by contrast, intriguing experimental and theoretical evidence has been put forward in recent years that suggests that coherent energy transport via short-lived superpositions of exciton states might contribute to the function of LHCs (*4*, *5*). Within this framework, electronic and/or vibronic coherences (*6*) enable delocalized excitons to move tens of nanometers on the time scale of hundreds of femtoseconds following photoexcitation before localization occurs. In the case of LHCs, such time scales are sufficient to allow efficient energy transfer from antennae complexes to reaction centers. However, for devices based on OSC films such as photovoltaics, photodetectors, and photocatalytic systems, the ideal length scales for exciton transport would be matched to the material’s absorption depth, typically 100 to 200 nm, and the coherent transport of energy over such distances is extremely challenging. Hence, energy transport in OSCs has remained largely restricted to FRET, and despite many decades of work, singlet exciton diffusion lengths (*L*_D_) in OSC films are typically limited to about 10 nm, with associated diffusion constants (*D*) on the order of 10^−3^ to 10^−4^ cm^2^/s (*3*, *7*).

The restriction to FRET/localized excitons in OSCs represents a substantial obstacle for devices, as the short *L*_D_’s lower device efficiencies and necessitate the use of nanoscale bulk heterojunctions, which compromise other properties such as charge extraction and stability. Improved energy transport has thus remained a long-outstanding goal. Promisingly, several recent observations have shown that, in isolated self-assembled nanostructures, exciton diffusion lengths and diffusion constants can be considerably higher (*8*–*10*). For example, oligomeric polyfluorene nanofibers (NFs) can show *L*_D_’s of 270 nm (*9*) and tubular porphyrin aggregates display *D* of 3 to 6 cm^2^/s (*10*). However, such behavior has not been extended to device-relevant films nor to materials with broad visible light absorption. Most critically of all, the fundamental question of how energetic and structural order can allow such a large increase in exciton mobility remains unanswered.

Here, we study films of well-ordered poly(3-hexylthiophene) NFs that absorb broadly across the visible spectral region and provide experimental and theoretical evidence for a new highly efficient energy transport regime in OSCs: transient delocalization. In this scheme, excitons still spend most of their lifetime in localized states as in FRET but retain access to delocalized states by exchanging energy with vibrational modes under equilibrium conditions, i.e., well after the initial (short-lived) period of delocalization following photoexcitation. We show that this allows the efficient transport of excitons over long distances at room temperature; in films of the NFs, we measure *D* to be 1.1 ± 0.1 cm^2^/s via ultrafast optical microscopy and spectroscopy, giving an estimated *L*_D_ of 300 ± 50 nm, which is higher than the absorption depth of the material.

## RESULTS

### Model OSC system

**Fig. 1:**
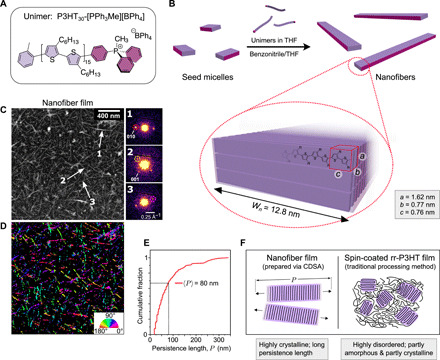
System of interest: rr-P3HT-based fiber-like micelles (i.e., NFs). (**A**) Unimer component of NFs. (**B**) Schematic of living CDSA of NFs, with an illustration of the unit cell calculated from powder x-ray diffraction. (**C**) Virtual annular dark-field scanning TEM micrograph of film of NFs with diffraction patterns at the marked points. (**D**) Corresponding orientation map. Color scale represents the orientation of the detected Bragg spots associated with the π-π stacking along the NF. (**E**) Corresponding cumulative distribution (per area) of the lengths of the continuous domains that exhibit the same orientation, i.e., the “persistence length.” The mean persistence length of 80 nm indicates that the chains in the NFs are highly aligned over large distances in the films. (**F**) Schematic of microstructure of NF films versus spin-coated rr-P3HT (a traditional processing method), highlighting how living CDSA results in a superior microstructure with alignment of polymer chains over large distances, while spin-coating rr-P3HT results in a mixture of amorphous and crystalline domains with limited long-range order.

Figure 1 displays the explored model system—regioregular poly(3-hexylthiophene) (rr-P3HT) NFs. To create the P3HT NFs, we use living crystallization-driven self-assembly (CDSA) for its ability to produce well-ordered, colloidally stable, and uniform nanostructures via the epitaxial crystallization of polymer-based amphiphiles, including those based on conjugated polymers (*11*–*16*). This seeded growth technique also allows exquisite size control, the formation of segmented assemblies from different building blocks, and surface functionalization, so its control and versatility makes it attractive for device fabrication (*9*, *17*–*20*). P3HT was chosen as the base material because it strongly absorbs visible light and has been widely studied for several decades as a model system for harvesting energy in organic photovoltaics, photodetectors, and photocatalysts. Using a recently described procedure, molecularly dissolved phosphonium borate–terminated P3HT amphiphile P3HT_30_-[PPh_3_Me][BPh_4_] in tetrahydrofuran (THF) was added to short seeds of the same material (see Fig. 1 and Materials and Methods). This yielded a sample of uniform fiber-like micelles (referred to as NFs) with an average length of 890 nm (length dispersity = 1.08). The NFs have a height of 4.5 nm as determined by atomic force microscopy (fig. S4), a width of 12.8 nm as determined by transmission electron microscopy (TEM), and a solvated corona corresponding to the phosphonium group at the terminus of polythiophene, which permits colloidal stability due to the introduction of electrostatic repulsion between the NFs and additional opportunities for solvation (*21*). The predominant NF component is a crystalline core that consists of the rr-P3HT segments (Fig. 1B). This crystalline rr-P3HT core has a well-defined crystal structure (as determined from x-ray diffraction measurements; see fig. S6), where the rr-P3HT chains pack side by side to afford good π-π orbital overlap and hence strong electronic interactions between neighboring chains. The NFs appear to be exclusively crystalline, as demonstrated by the homogeneous profile observed in both TEM (Fig. 1C and fig. S5) and confocal laser scanning microscopy (fig. S14), as well as the lack of photoluminescence (PL) that would normally be attributed to amorphous chains (see fig. S23 for more details).

We prepare films from the NFs (see Materials and Methods) to produce dense meshes of NFs. To characterize these films, we use low-dose scanning electron diffraction (SED), which gives Bragg diffraction spots corresponding to the π-π stacking of the NFs with a ~4-nm resolution (see Fig. 1C). By analyzing the angle of the diffraction spots, an “orientation” map can be formed (Fig. 1D), where the color records the angle of the pair of diffraction spots, i.e., the orientation of the π-π stacking planes. While the NFs are mostly orientated randomly with respect to one another (as indicated by the different overall colors of each NF), the NFs on their own tend to exhibit large straight sections with a consistent color, indicating that the rr-P3HT chains are fully aligned over these domains. To parameterize the length of these straight, aligned domains, we introduce the persistence length, *P* (see the Supplementary Materials for more details and fig. S9 for an illustration). The cumulative distribution of the extracted *P*’s in Fig. 1E shows that the NFs tend to exhibit large persistence lengths, with a mean value of 〈*P*〉 = 80 nm and detected lengths up to *P* > 300 nm. This establishes excellent long-range ordering of rr-P3HT chains in the NFs when in the films, a consequence of the epitaxial growth mechanism inherent to living CDSA. The measurement of 〈*P*〉 is limited in this case by out-of-plane bending of the NFs in the films, which introduces deviations from the Bragg condition.

**Fig. 2:**
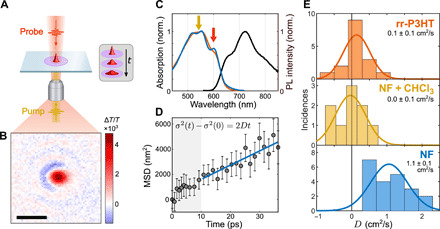
Transient absorption microscopy of NF film and controls. (**A**) Schematic of TAM experiment with emphasis on expansion of Gaussian profile due to exciton diffusion. (**B**) Example of Δ*T*/*T* image close to time zero measured at 600 nm. (**C**) Normalized absorption (blue) and steady-state PL spectra (black) of NF film. The absorption of a spin-coated rr-P3HT film (orange) matches the NFs’ absorption due to the identical π-π chain packing motif. The yellow and red arrows indicate the central wavelengths of the pump (540 nm) and probe (600 nm), respectively. (**D**) Typical MSD profile at an average fluence of 4 μJ/cm^2^ with a linear fit to the region from 10 to 30 ps to extract *D*. At this later region, there is no observed dependence of *D* with fluence. (**E**) Histograms of *D* from 10 to 30 ps for multiple measurements on a control spin-coated rr-P3HT film (rr-P3HT), control NFs treated with chloroform to destroy their long-range order (NF + CHCl_3_), and the pristine NF film. Insets are mean value of *D* in each case. The NFs exhibit a large diffusion constant of *D*_NF_ = 1.1 ± 0.1 cm^2^/s, while the controls both show behavior consistent with a small diffusion constant (<0.1 cm^2^/s). The clear difference between the NFs and controls highlights how the NFs’ large exciton diffusivity is a function of their superior morphology/energetic order. Note that the spread in the values of *D* are primarily due to random error. Uncertainties are the SD over $\sqrt{N}$, with *N* being the number of measurements.

The NFs’ long-range structural order contrasts with that which is typically observed in polymer films, including self-organizing high–molecular weight spin-coated rr-P3HT (herein referred to as “rr-P3HT”), which has been studied extensively for over two decades. Although both the NF films and rr-P3HT exhibit the same π-π stacking motif (*22*)—exemplified in Fig. 2C by the similar absorption profile—Fig. 1F illustrates how rr-P3HT consists of both amorphous and crystalline regions, with a high degree of energetic and structural disorder, and limited long-range ordering of polymer chains (*22*, *23*).

### Direct measurement of efficient energy transport

We use transient absorption microscopy (TAM) to directly image the interchain exciton diffusion along the NFs in the NF films. As shown in Fig. 2 (A and B), this technique uses wide-field imaging via a time delayed ~100-fs probe pulse, following excitation with a diffraction-limited ~30-fs pump pulse centered at 540 nm [see (*24*, *25*) for further information]. We measure a differential transmission distribution (Δ*T*/*T*) at a probe wavelength of 600 nm as a function of pump-probe delay (see the Supplementary Materials and Fig. 2 for further details). Because the Δ*T*/*T* distribution is proportional to the photogenerated exciton distribution, *n*(*t*, *x*, *y*), expansion of the Δ*T*/*T* distribution (see inset in Fig. 2A) is related to the movement of excitons. This movement can be parameterized by the mean square displacement, MSD(*t*), of the ensemble, which is defined by the following: MSD(*t*) ≡ σ^2^(*t*) − σ^2^(0), where σ^2^(*t*) is the spatial variance of the exciton distribution extracted by fitting a two-dimensional (2D) Gaussian to each Δ*T*/*T* image.

Figure 2D plots the MSD(*t*) for a representative measurement. We note that, at early times, we observe signatures of exciton-exciton annihilation (EEA) at the fluence used here (4 μJ/cm^2^), which would result in an overestimation of *D* at early times. However, for time delays beyond 10 ps, the extracted *D* values are not affected by pump fluence (see fig. S27 and detailed discussion in the Supplementary Materials). It can be seen in Fig. 2D how MSD(*t*) is linearly related to time in this region, allowing a reliable extraction of *D* [MSD(*t*) = 2*Dt*; see the Supplementary Materials for details]. Performing many such individual measurements across different locations on different films allows us to build up statistics for *D*, as shown in Fig. 2E. Together, the average diffusion constant is *D*_NF_ = 1.1 ± 0.1 cm^2^/s.

To rule out systematic artefacts, we also performed measurements on two control samples: rr-P3HT films and films of chloroform (CHCl_3_)–treated NFs (where application of CHCl_3_ on the film disrupts the long-range order of the NFs; see Materials and Methods and fig. S12 for details). The resultant diffusion constants were both below the experimental detection limit of about 0.1 cm^2^/s. For rr-P3HT, this is expected; *D* is known to be significantly below 0.1 cm^2^/s for solution-processed films (*26*, *27*). Furthermore, a low value of *D*_NF + CHCl_3__ is also to be expected if *D*_NF_ is high because of the NF’s intrinsic order. We are therefore able to rule out any systematic artefacts contributing to *D*_NF_.

We also analyzed EEA effects in femtosecond transient absorption spectroscopy (fs-TA) and time-resolved PL (TRPL) to estimate *D*_NF_ (see the Supplementary Materials for full details). In these measurements, we (indirectly) find that *D* ranges from 0.2 to 0.8 cm^2^/s for fs-TA and 0.2 to 1.0 cm^2^/s for TRPL, which broadly agree with the more accurate *D*_NF_ value directly found using TAM.

Our experimental data therefore establish a diffusion constant of *D*_NF_ = 1.1 ± 0.1 cm^2^/s in the NF films. This value is remarkable considering that previous reports of exciton diffusion within crystalline regions of rr-P3HT gave *D* as 2 × 10^−2^ cm^2^/s (*28*), 7.9 × 10^−3^ cm^2^/s (*27*), and 1 × 10^−2^ cm^2^/s (*26*), and most reported values for OSC films are in the range of 10^−3^ to 10^−4^ cm^2^/s (*3*, *27*, *29*). Notably, this *D*_NF_ value is found for an OSC film—which is most relevant for devices—as opposed to previous observations of efficient (equilibrium) transport that were found for isolated and pristine single nanostructures (*8*–*10*). Combined with the exciton lifetime of τ = 400 ± 100 ps extracted using fs-TA (see fig. S19), we estimate a corresponding diffusion length of $L_{\text{NF}}=\sqrt{2D\tau }=300\pm 50$ nm. This calculation of *L*_NF_ assumes that *D* remains constant over the exciton’s lifetime, which is a reasonable assumption as we detail later. This value is an order of magnitude higher than previously reported values for rr-P3HT of 20 and 27 nm (*26*, *27*) or any solution-processed conjugated polymer film.

### Energetic disorder

**Fig. 3:**
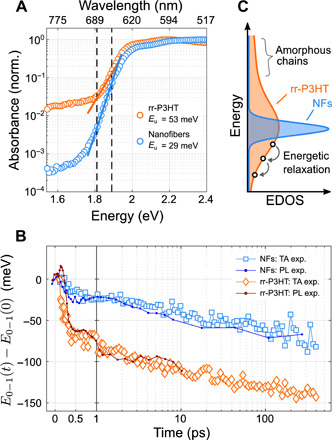
Energetic order in NF films versus spin-coated rr-P3HT. (**A**) PDS reveals that the NFs have an Urbach energy, *E*_u_, of 29 ± 1 meV, almost half that of the spin-coated rr-P3HT films, indicating that the NFs are much more energetically ordered. The NFs also have, on average, over 40× less absorption of low-energy (<1.7 eV) trap states. *E*_u_ is found by linear fitting to the regions enclosed by the dashed lines. (**B**) Red-shifting of 0 to 1 vibronic emission peak (see the Supplementary Materials for fitting details) independently measured by two techniques: fs-TA and TRPL. Red shifting indicates relaxation in a disordered EDOS, and accordingly, there is substantially less relaxation in the NFs in comparison to rr-P3HT. (**C**) Schematic of EDOS after photoexcitation, illustrating how that, in direct contrast to rr-P3HT, NFs have a tight EDOS due to energetic order, which inhibits the energetic relaxation of excitons. This restricted relaxation promotes the spatial diffusion of excitons, as neighboring sites are more likely to be thermally accessible.

In any mechanistic picture of exciton transport, energetic disorder due to local imperfections or variations in the crystalline packing of molecules or polymer chains is expected to restrict exciton diffusion because excitons may get trapped at low-energy sites (*2*, *3*, *7*, *30*–*33*). We hence characterize the energetic order of the NF and rr-P3HT films. Using photothermal deflection spectroscopy (PDS), we extract an Urbach energy (*E*_u_)—the width of the sub-bandgap tail states—of *E*_u; NF_ = 29 ± 1 meV for the NF films (see Fig. 3A). This value is significantly lower than the value we find for rr-P3HT (53 ± 6 meV), in agreement with literature (*34*). To our knowledge, *E*_u; NF_ is the lowest value measured for a P3HT-based system, and among the lowest reported for any OSC (*35*–*37*). We also note that the NF’s baseline absorption at low energies (<1.7 eV) is more than 40 times lower than that of rr-P3HT, indicating a greatly reduced density of deep trap states. This lack of deep traps in the NFs is understandable, given the living CDSA growth mechanism, because the slow epitaxial growth from the seed micelles will tend to exclude chains with defects, prevent twists or kinks in the chains, and overall result in much purer crystal than spin-coating.

To examine the effect of this energetic disorder on the relaxation of excitons within the excitonic density of states (EDOS) (which occurs via exciton diffusion to lower-energy sites), we use a combination fs-TA and TRPL. As shown in Fig. 3B, in the NF films, the position of the *E*_0 − 1_(*t*) vibronic peak relaxes by only ~20 meV over 1 ps following photoexcitation. In contrast, excitons in rr-P3HT rapidly relax ~80 meV by 1 ps. The fact that this value (20 meV) and the Urbach energy (29 meV) are comparable to *k*_B_*T* at room temperature (26 meV) suggests that thermal fluctuations can easily overcome energetic disorder within the NF films and that excitons do not become deeply trapped. We also highlight that the average *E*_0 − 1_ position in TRPL changes only 6 meV between the time ranges of 10 to 30 ps and 30 to 375 ps (see fig. S24), and so the *D*_NF_ value extracted from 10 to 30 ps is expected to be valid at later times, lending validity to our estimate of *L*_NF_ previously.

### Exciton transport mechanism

We now examine the mechanistic nature of the exciton transport. One possibility is the classical FRET-based picture, where localized excitons hop from site to site. This is the predominant description given for exciton transport in OSC films. However, in our case, we find that a simple Förster hopping model compares very poorly with experiment, giving an upper value of *D*_NF_~2 × 10^−3^ cm^2^/s (a value that is similar to those given in previous reports; see the Supplementary Materials for more details) (*28*, *31*, *38*). Alternatively, one might speculate that the NFs’ behavior is similar to that proposed in LHCs, where short-lived delocalized states at early times are mainly responsible for the exciton transport. Again, however, this picture does not fit the experimental data; we observe no anomalous transport at early times, and *D*_NF_ is extracted from 10 ps onward, by which time excitons will have reached quasi-equilibrium conditions (as shown by the data in Fig. 3B).

**Fig. 4:**
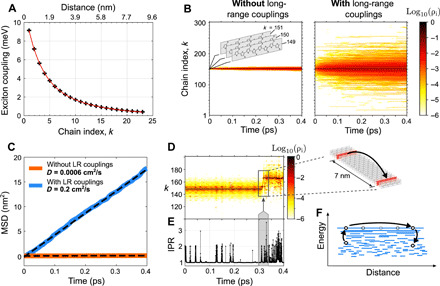
Simulations of exciton diffusion. (**A**) INDO/SCI (intermediate neglect of differential overlap/single configuration interaction) calculations of the Coulombic exciton couplings in a 24-chain stack, with a biexponential fit. (**B**) Sum of many individual surface hopping simulations of exciton diffusion in a 1D stack (i.e., trajectories) with and without long-range (LR) exciton couplings. The color scale indicates the log of the normalized total exciton density. (**C**) Associated MSD derived from (B). The MSD evolves linearly, indicating that an equilibrium regime is reached. Linear fits (dashed) give diffusion constants of *D* = 0.22 cm^2^/s and 6 × 10^−4^ cm^2^/s with and without the inclusion of long-range exciton couplings, respectively, which demonstrates how long-range couplings markedly increase diffusion. (**D**) Exciton density over a single representative trajectory, with (**E**) the associated IPR. The IPR remains mostly low (~1), indicating that the exciton is mostly localized on a single chain, but occasionally (as indicated in the highlighted region), the IPR increases as the exciton transiently occupies higher-energy delocalized states, and when it does, the exciton tends to move large distances by “surfing” along the EDOS as shown by the inset. (**F**) Schematic illustrating how excitons can transiently occupy high-energy delocalized states to move large distances. These delocalized states are responsible for the majority of the diffusion, a fact that is emphasized by making the comparison to the case of no long-range couplings, where no such delocalized states are occupied, and the diffusion is severely restricted.

To model the time-dependent behavior of the exciton wave function and hence model exciton transport, we use nonadiabatic molecular dynamics simulations. The diffusion of excitons was simulated in 1D stacks of ~300 P3HT chains as shown in Fig. 4B using a mixed quantum-classical, crossing-corrected variant of the subspace surface hopping algorithm (*39*–*41*), which incorporates stochastic nonadiabatic transitions between different adiabatic potential energy surfaces (PESs) (*42*). The inclusion of long-range exciton couplings in the simulation is physically realistic, given the mean persistence length of 〈*P*〉=80 nm as measured by SED. The time-evolved exciton wave functions are calculated from 10,000 individual trajectories and are then averaged to compute an ensemble exciton density and MSD(*t*) (see Fig. 4, B and C). The corresponding diffusion constant of *D*_sim_ = 0.22 cm^2^/s is the same order of magnitude as our experimental value of *D*_NF_ = 1.1 ± 0.1 cm^2^/s.

To interrogate the reason for such a high *D*_sim_ value, we assess the role of exciton delocalization. We compute the inverse participation ratio (IPR)—a proxy for the interchain exciton delocalization—and find a mean IPR of ~1 (see the Supplementary Materials), demonstrating that excitons are mostly localized on a single chain. However, analysis of the time-dependent IPR profile of the excitonic wave function over a single randomly selected trajectory (see Fig. 4, D and E) reveals something curious: While the excitation remains mostly confined over a single chain at the bottom of the EDOS, it occasionally momentarily reaches slightly higher-energy adiabatic states that spread over several neighboring chains. This transient access to delocalized states occurs because of the continuous energy exchange with vibrational modes in the material, serving to kick the exciton over large distances on a short time span. As shown schematically in Fig. 4F, the exciton sporadically “surfs” on the EDOS. We propose that this transient delocalization enables the large values of *D* experimentally measured here. We note that the proposed model is similar to the mobility edge model of charge transport (*43*) in OSCs, and a similar scenario has been shown to accurately reproduce charge mobilities in organic crystals (*44*).

This “transient delocalization” scenario is critically dependent on the presence of long-range exciton couplings; as shown in Fig. 4 (B and C), when all non-nearest neighbor interactions are set to zero (thus adopting the tight-binding approximation used for charges), there is no transient access to delocalized states, which reduces *D* by three orders of magnitude to *D* = 6 × 10^−4^ cm^2^/s, which is similar to the value for *D* previously obtained in rr-P3HT films (*3*, *26*, *27*, *29*, *45*). We note that stacking faults, point defects, or grain boundaries would shorten the effective range of the excitonic interchain interactions, thereby deactivating transient delocalization. Crucially, this is avoided in our case, with multiple techniques demonstrating high crystallinity in the NF films, and the long persistence length of 80 nm observed in SED confirming the existence of long-range excitonic interchain interactions. This highlights the advantage of our highly controlled living CDSA synthetic process in enabling long-range structural order and hence transient delocalization.

On the other hand, *D* is only moderately sensitive to energetic disorder; when the inhomogeneous broadening is halved or doubled, the extracted *D* only increases or decreases by ~40%, respectively (see fig. S29), suggesting that long-range interactions smooth out, to some extent, energetic inhomogeneity in the material (*35*). A key point may be the absence of deep energetic traps, as indicated by the PDS data, in comparison to conventional materials. Once trapped, in such sites, the wave function would not be able to access the delocalized states via energy exchange with vibrational modes.

## DISCUSSION

In summary, we have demonstrated that well-ordered visible light–absorbing films of P3HT NFs prepared via living CDSA exhibit unprecedented exciton diffusion properties, and to explain these results, we propose a new energy transport framework: transient delocalization. In this new scheme, the lowest-energy exciton state is still highly localized (as is the case for most OSCs); however, energy exchange with vibrational modes can temporarily promote excitons to delocalized states, with this transient access responsible for a two- to three-order-of-magnitude enhancement in the energy transport rate. This new picture challenges the widely held notion that excitons in OSCs are localized under equilibrium conditions; instead, they can experience large fluctuations in their spatial extent, given the right structural and energetic conditions, with briefly occupied delocalized states playing an unexpectedly large role in the transport process. This highly efficient regime of energy transport should be achievable across a wide range of device-relevant OSCs, as is the case for the equivalent charge transport mechanism (transient charge delocalization/localization). We expect further exploration of the emergent transient delocalization behavior to prove highly fruitful, with particularly interesting directions including its temperature dependence, its dependence on the unimer’s length, and the inclusion of nonlocal electron-phonon couplings into the model. This may help reveal further design rules in addition to our two foundational rules that energetic disorder be comparable to *k*_B_*T* so that energy exchange with vibrational modes may provide access to extended states, and long-range structural order over tens of nanometers so that these extended states can be supported. Engineering transient delocalization in devices may thus provide a route toward improved efficiencies in a range of OSC devices, particularly because the crystalline order that transient delocalization demands would also be beneficial for properties such as charge extraction.

Our work also demonstrates the power of living CDSA for engineering efficient transport in visible light–absorbing thin films. This may help improve the performance of conventional devices and, interestingly, opens up new device architectures for photovoltaics, photodetectors, photocatalytic systems, and photon up/down-conversion systems that would no longer be limited by the nanoscale bulk heterojunction architecture and could funnel energy over hundreds of nanometers to active sites/heterojunctions. For example, recent advances in living CDSA have demonstrated the ability to grow nanostructures directly off silicon surfaces (*19*). This could allow semiconducting inorganic-organic heterojunctions where high exciton diffusivity in conjunction with strong visible light absorption could be exploited for enhanced photodetection.

## MATERIALS AND METHODS

### Preparation of P3HT NFs via living CDSA for the preparation of films

The solution of the seed micelles (concentration = 0.5 mg/ml) in benzonitrile/THF mixture (benzonitrile:THF = 8:2 v/v) was warmed at 35°C for 5 min. Appropriate amounts of unimers in THF were added to the seed micelles. After the mechanical shaking for 10 s, the solutions were aged for 12 hours. THF and some benzonitrile were evaporated by a gentle N_2_ gas flow for 3 hours (final concentration = 1.0 mg/ml). TEM analysis was used to evaluate the length of the resulting micelles after dilution to 0.02 mg/ml using benzonitrile.

### Preparation of films for spectroscopy

All film preparation was conducted in an N_2_ atmosphere. Unless otherwise stated, the samples were deposited on 22 mm × 22 mm No. 1.5 borosilicate 170-μm-thick glass coverslips (Deckgläser), which were precleaned via 10-min sonication in acetone/water = 8:2 (v/v) and then isopropanol. The films were subsequently encapsulated in the same N_2_ glove box by placing a second smaller coverslip atop the sample substrate with a ~200-μm-thick carbon tape spacer and sealing with epoxy resin. All spectroscopy experiments were performed at room temperature.

The NF films used for spectroscopy were prepared via drop-casting to produce films consisting of a dense, randomly orientated mesh of NFs stacked atop each other. Each film was allowed 12 hours for the benzonitrile solvent to fully dry. These NF films were all prepared from the same original batch of NFs. The rr-P3HT films were made via spin-coating solutions of rr-P3HT (96% regioregularity, average molecular weight of 90 kDa, and polydispersity index of 2.3) in chlorobenzene at 2000 rpm. The chloroform-treated NF films were prepared by first dissolving the NFs in CHCl_3_. The resultant unimer solution was drop-casted onto a glass slide heated to ~80°C to rapidly remove the solvent, thereby preventing any substantial long-range crystallization. CHCl_3_ was then reapplied to the film to dissolve the unimers a second time so that the solvent was now exclusively CHCl_3_ and was then rapidly dried again. All the films were optimized for spectroscopy so that their absorption peaks (which all match up very well; see the Supplementary Materials) corresponded to ~0.3 OD (optical density) in the visible region.

### Scanning electron diffraction

Samples were prepared by drop-casting the NFs onto carbon-coated copper grids (Agar Scientific). SED data were acquired using a JEOL ARM300CF microscope fitted with an ultrahigh-resolution pole piece, a cold field emission gun, and aberration correctors in both the probe-forming and image-forming optics (Diamond Light Source, UK). The instrument was operated at 300 kV. A nanobeam configuration was obtained by switching off the aberration corrector in the probe-forming optics and using a 10-μm condenser aperture to obtain a convergence semiangle <1 mrad and a diffraction-limited probe diameter of ca. 3 nm. The probe current was measured using a Faraday cup as ca. 2 pA, and the exposure time was 1 ms per probe position. The estimated electron fluence, assuming a disk-like probe, was <20 e^−^ Å^−2^. A diffraction pattern was acquired at every probe position using a quad-chip Merlin Medipix hybrid counting-type direct electron detector (Quantum Detectors, UK) with 512 × 512 pixels. SED was obtained in a “blind scanning” point-and-shoot workflow to minimize the total electron fluence the specimen received.

SED data were processed using pyxem-0.11.0 (*46*). The diffraction patterns were aligned and calibrated following previously reported procedures (*47*). Briefly, the calibration of the scan step size and the diffraction pattern pixel size was performed using a standard 500-nm gold diffraction grating replica with latex spheres (Ted Pella). The cross-grating data were also used to determine residual elliptical distortions of the diffraction patterns due to the post-specimen optics. The rotation between the diffraction pattern and the real-space orientation was calibrated using an MoO_3_ standard (Agar Scientific). Processing of the diffraction patterns included the following: (i) centering the direct beam in each diffraction pattern within the data array using a cross-correlation routine and (ii) applying an affine transformation to correct for elliptical distortion and rotation between the real-space scan and the diffraction pattern, as determined by calibration.

### Photothermal deflection spectroscopy

PDS sensitively measures absorption directly by probing the heating effect in samples upon absorption of light. Films were coated on a Spectrosil fused-silica substrate and were immersed in an inert liquid FC-72 Fluorinert. They were then excited with a modulated monochromated light beam perpendicular to the plane of the sample. A combination of a Light Support MKII 100W Xenon arc source and a CVI DK240 monochromator was used to produce a modulated monochromated light beam. The PDS measurements were acquired by monitoring the deflection of a fixed wavelength (670 nm) diode laser probe beam following absorption of each monochromatic pump wavelength.

### Transient absorption microscopy

A thorough description of the setup can be found in (*23*), but in brief, a Yb:KGW-based amplified laser system (PHAROS, Light Conversion) provided 200-fs, 30-μJ pulses at 1030 nm with a 200-kHz repetition rate. The output beam was split to seed two broadband white-light continuum (WLC) stages. The probe WLC was generated in a 3-mm yttrium-aluminum-garnet (YAG) crystal and adjusted to cover a 580- to 950-nm range via a fused-silica prism-based spectral filter. The pump WLC was generated in a 3-mm sapphire crystal to extend the WLC to 500 nm and short-pass–filtered at 575 nm. A set of chirped mirrors (2× for pump) and a pair of fused-silica wedges were used to precompress the pulses to ~100 fs (probe) and ~30 fs (pump). Shorter probe pulses (~10 fs) can be achieved by reducing the bandwidth; however, this ultrafast time resolution was not needed, and the priority here was instead to probe at 600 nm (which corresponds to a ground-state bleach in the samples analyzed). A motorized delay stage (Newport) was used to delay the probe with respect to the pump. A clean mode for the pump was achieved with a pinhole. The pump was collimated to completely fill the aperture of the objective lens (×100, with an effective numerical aperture of 1.1) to deliver a near diffraction-limited spot with a ~270-nm full width at half maximum (FWHM). The probe was counter-propagated through the sample with a relatively large focal spot (~15 μm). The pump and probe polarizations were set to be parallel at the sample to maximize signal-to-noise. The transmitted probe pulse was collected by the same objective lens, was filtered with a 600-nm bandpass filter (10-nm FWHM), and imaged onto an electron-multiplying charge-coupled device (CCD) camera (Rolera Thunder, QImaging). An automatic focus control loop based on total internal reflection of a reference continuous wave laser (405 nm) was used to stabilize the focus position via an objective piezo (NP140, Newport). Differential imaging was achieved by modulating the pump beam at 30 Hz with a mechanical chopper.

### Transient absorption spectroscopy

The transient absorption experiments were performed using a Yb:KGW laser system (PHAROS, Light Conversion) to provide 15.2 W at 1030 nm with a 38-kHz repetition rate. The probe beam was generated by focusing a portion of the fundamental in a 4-mm YAG crystal to generate a WLC. The pump beam was generated by a noncollinear optical parametric amplified (NOPA) seeded by WLC from a 3-mm YAG crystal mixed with a third harmonic pump (HIRO, Light Conversion) in a barium borate crystal (37° cut, type I, 5° external angle). The NOPA output was centered at 540 nm and then compressed down to ~10-fs pulses using a pair of chirped mirrors and a pair of fused-silica wedges. The pump was delayed using a computer-controlled Thorlabs translation stage. A sequence of probe pulses with and without a pump was generated using a chopper wheel on the pump beam. After the sample, the probe pulse was split with a 950-nm dichroic mirror (Thorlabs). The visible part was then imaged with a Silicon photodiode array camera (Stresing Entwicklunsbüro; visible monochromator 550-nm blazed grating). The near-infrared portion of the probe was seeded to an infrared spectrograph (1200-nm blazed grating) and imaged on an InGaAs photodiode array camera (Sensors Unlimited). Offsets for the differing spectral response of the detectors were accounted for in the post-processing of data.

### Transient gating PL spectroscopy

For the detection of the broadband PL on a subpicosecond time scale, we used the transient grating PL technique (*48*). The output of a femtosecond ytterbium fiber laser (Tangerine SP, Amplitude Systems) operating at 44-kHz pulses was split into pump and gate parts. The pump part was frequency-doubled to 515 nm by using a beta barium borate crystal and focused to a 62-μm^2^ spot to excite the samples. The PL was collimated and refocused onto the gate medium (2-mm undoped YAG crystal) using a pair of off-axis parabolic mirrors. For the gate part, ~40-μJ, 1030-nm output was split using a 50:50 beam splitter to generate the two gate beams and focused onto the gate medium at a crossing angle of approximately 8° and overlapped with the PL in a BOXCAR geometry. The two gate beams generate a laser-induced grating inside the gate medium, acting as an ultrafast gate to temporally resolve the broadband PL signals by diffracting the gated signal from the PL background. Two achromatic lenses collimated and focused the gated signals onto the spectrometer entrance (Princeton Instruments SP2150), and the gated PL spectra were measured by an intensified CCD camera (Princeton Instruments, PIMAX3). Long- and short-pass filters were used to remove the residual pump and intense 1030-nm gate, respectively. The time delay between pump and gate beams was controlled via a motorized optical delay line. For the transient PL spectrum at each time delay, 120,000 shots were accumulated. The setup can achieve detection bandwidth from 530 to 900 nm, limited by the color filter pair and instrument response function (~230 fs).

### Theory

We model the steady-state optical properties of 24-mer aggregates applying a Frenkel-Holstein model (solved in the two-particle approximation and with open boundary conditions). The Coulomb excitonic couplings are calculated using a transition density approach based on semi-empirical quantum chemical calculations of the isolated polymer chains. Couplings of the electronic excitations to a high-frequency vibration at 0.18 eV (1450 cm^−1^) and to low-frequency vibrations are explicitly included to reproduce the measured vibronic progression in optical absorption. The excitation energies are sampled from a noncorrelated Gaussian shape inhomogeneous disorder distribution. Under the (reasonable) assumption that steady-state PL occurs from the thermalized EDOS, i.e., in equilibrium conditions, simultaneously fitting the line shape and linewidth in emission and the spectral Stokes shift yield the magnitude of the static (over the time scale of energy migration) and dynamic contributions to the energy disorder.

The same Hamiltonian is then used to model the propagation of the excitons along the 1D stacks using a mixed quantum-classical, crossing-corrected variant of subspace surface hopping algorithm (*39*–*41*), which incorporates stochastic nonadiabatic transitions between different adiabatic PES (*42*). The dynamics of the full system is described by an ensemble of independent trajectories, where each trajectory occupies an “active” PES at individual time steps (see details of the methodology in the Supplementary Materials). Along each trajectory, the dynamics of the ions is treated classically by the Langevin equations of motion, while the exciton wave function is propagated quantum-mechanically solving the time-dependent Schrödinger equation. The time-evolved exciton wave function is then used to calculate the MSD and the IPR, the latter providing a proxy for the intermolecular exciton delocalization. Linear evolution of MSD signifies that an equilibrium diffusion regime is attained, out of which the diffusion coefficient (*D*) can be calculated. To study the nonadiabatic excited-state dynamics, we consider a stack consisting of ~300 rr-P3HT chains (with open boundary conditions), initially exciting the central chain of the stack.

## SUPPLEMENTARY MATERIALS

Supplementary material for this article is available at http://advances.sciencemag.org/cgi/content/full/7/32/eabh4232/DC1
